# Colonic Endometriosis Mimicking Colon Cancer on a Virtual Colonoscopy Study: A Potential Pitfall in Diagnosis

**DOI:** 10.1155/2009/379578

**Published:** 2009-11-08

**Authors:** Jonathan D. Samet, Karen M. Horton, Elliot K. Fishman, Ralph H. Hruban

**Affiliations:** ^1^Department of Radiology, Northwestern Medical Faculty Foundation, Chicago, IL 60611, USA; ^2^Department of Radiology, Johns Hopkins Medical Institutions, Baltimore, MD 21287-0801, USA; ^3^Department of Pathology, Johns Hopkins Medical Institutions, Baltimore, MD 21231, USA

## Abstract

Colonic endometriosis has been reported in the literature to mimic colon cancer. Patients can present with symptoms almost identical to colon cancer. We present an exemplary case of a woman who was found to have a mass on conventional colonoscopy. Virtual colonoscopy was instrumental in characterizing the obstructive sigmoid mass. A biopsy of the mass revealed sigmoid endometriosis.

## 1. Introduction

Colonic endometriosis mimicking adenocarcinoma of the colon is a puzzling scenario for the clinician. Case reports are scattered throughout the literature describing this phenomenon [1–7]. The patient's symptoms can be similar to colon cancer, with rectal bleeding, change in bowel habits, and even bowel obstruction. We present a case of a woman referred for virtual colonoscopy after a conventional colonoscopy revealed an obstructing sigmoid mass, a presumed colon cancer. Subsequent biopsy and surgical pathology revealed endometriosis.

## 2. Case Report

The patient is a 51-year-old female who presented with a several week history of change in bowel habits, an episode of blood per rectum, and abdominal pain. She denied any weight loss, or change in appetite. Her past medical history was significant for a remote history of endometriosis with infertility and chronic hepatitis B infection. She underwent elective outpatient colonoscopy, which revealed a sigmoid colon mass causing marked stenosis. The colonoscopist was unable to pass the lesion. Biopsies were taken. The patient was then sent down to CT for a virtual colonoscopy to better evaluate the mass and also to image the remainder of the colon as well as to stage the presumed colon cancer. Given the high suspicion of colon cancer, the virtual colonoscopy was performed using IV contrast. Despite the remote history of endometriosis, it was not initially considered in the differential diagnosis.

The virtual colonoscopy revealed an apple-core lesion in the sigmoid colon as well as a segment of thickening and nodularity in the left colon. The biopsies taken during the colonoscopy revealed endometriosis. Seven days later, the patient was taken to surgery due to the obstruction. A 3 cm mass was found in surgery that was adherent to the bladder and the sigmoid colon. The specimen was sent to pathology which confirmed that it was endometriosis with hyperplasia involving the full thickness of the bowel wall with no evidence of malignancy. Of note, there were several benign lymph nodes, one with associated endometriosis.

## 3. Discussion

Endometriosis is defined as the aberrant location of endometrial tissue. It has a variably reported prevalence due to the difficulty of diagnosis and select groups that are studied. Estimates range from about 5 to 10% of woman of child bearing age [8]. Because endometrial tissue is fed by ovarian hormones, endometriosis is rare in pre- and postmenopausal women, but there are reports of it [9]. When looking at woman undergoing major gynecologic surgery for various reasons, 1% was found to have endometriosis. When looking at women who undergoes laparoscopy for chronic pelvic pain, the frequency increases to 12–32% [10–12].

The pathogenesis of endometriosis has been controversial. The implantation theory says that endometrial tissue that is shed during menstruation refluxes into the fallopian tubes and occasionally out into the peritoneum. This theory fits with the fact that the implants closest to the uterus are more common; whereas, the implants in other organ systems are rare.

Endometriosis is often classified as either pelvic or extrapelvic in location. In patients with pelvic endometriosis, endometrial tissue is confined to the fallopian tubes, ovaries, and nearby pelvic peritoneum [8]. The classic triad of symptoms is dyspareunia, dsymenorrhea, and infertility. Symptoms are said to relapse and remit in accordance with the menstrual cycle, but this only occurs in about 40% of patients [8]. Extrapelvic endometriosis can be located virtually anywhere else in the body, including involvement of the intestines, urinary system, skin, brain, muscles, lungs, liver, gallbladder, and even the heart [8]. The vast majority of extrapelvic endometriosis is in the rectum and sigmoid colon, accounting for about 95% [8]. The median age at the time of diagnosis is between 34 and 40 years [8], with up to seven percent of intestinal endometriosis reported in postmenopausal women [13]. Our patient presented to us at 51 years old with symptomatic sigmoid endometriosis. She was perimenopausal at the time of diagnosis. Intestinal implants of endometrial tissue are usually asymptomatic and clinically not worrisome. When intestinal endometrial lesions sometimes do cause symptoms, there can be wide variety of gastrointestinal symptoms including, dyschezia, blood per rectum, constipation, vomiting, diarrhea, and abdominal pain.

Diagnosis of intestinal endometriosis is difficult and as demonstrated by this case and others can be confused with other more serious lesions such as colon cancer, but there are a few defining characteristics. By nature of its proposed evolution, endometrial tissue usually involves the outer walls of the colon such as the serosal layer or submucosa. A lesion that penetrates the mucosa is less likely to be an endometrial lesion [14, 15]. For this reason, colonoscopy can easily miss a colonic endometrioma. However, in our case, the full thickness of the bowel wall was involved. Because the lumen of sigmoid bowel was so narrowed in our patient, a complete conventional colonoscopy examination was impossible. Virtual colonoscopy helped us describe the lesion more fully and visualize the part of the colon proximal to where the endoscope could not pass. However, the CT colonoscopy was ordered to evaluate the remainder of the colon and to fully characterize the presumed obstructing colon cancer. Had endometriosis been considered as a potential cause of the colonoscopy findings, MRI would have been a better modality to potentially characterize endometrial implants, while awaiting the biopsy results. CT or CT colonoscopy is not sensitive or specific in evaluating patients with endometriosis.

In conclusion, endometrial implants in the colon can present clinically identical colon cancer and should probably have been considered as a potential etiology of the findings on conventional colonoscopy. While endometriosis is a difficult radiologic diagnosis to make, it must be considered in women being worked up for a colon mass when the clinical picture is unclear. However, biopsy is usually necessary to confirm the diagnosis, as endometriosis can mimic colon cancer both on the conventional colonoscopy and virtual colonoscopy, as in this paper.

## Figures and Tables

**Figure 1:**
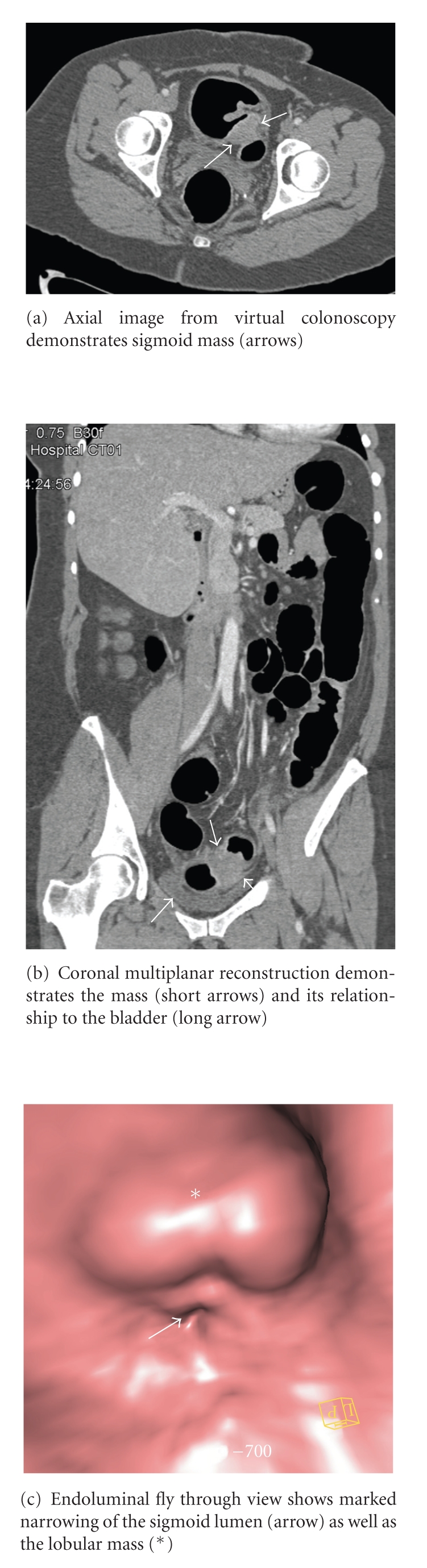


**Figure 2:**
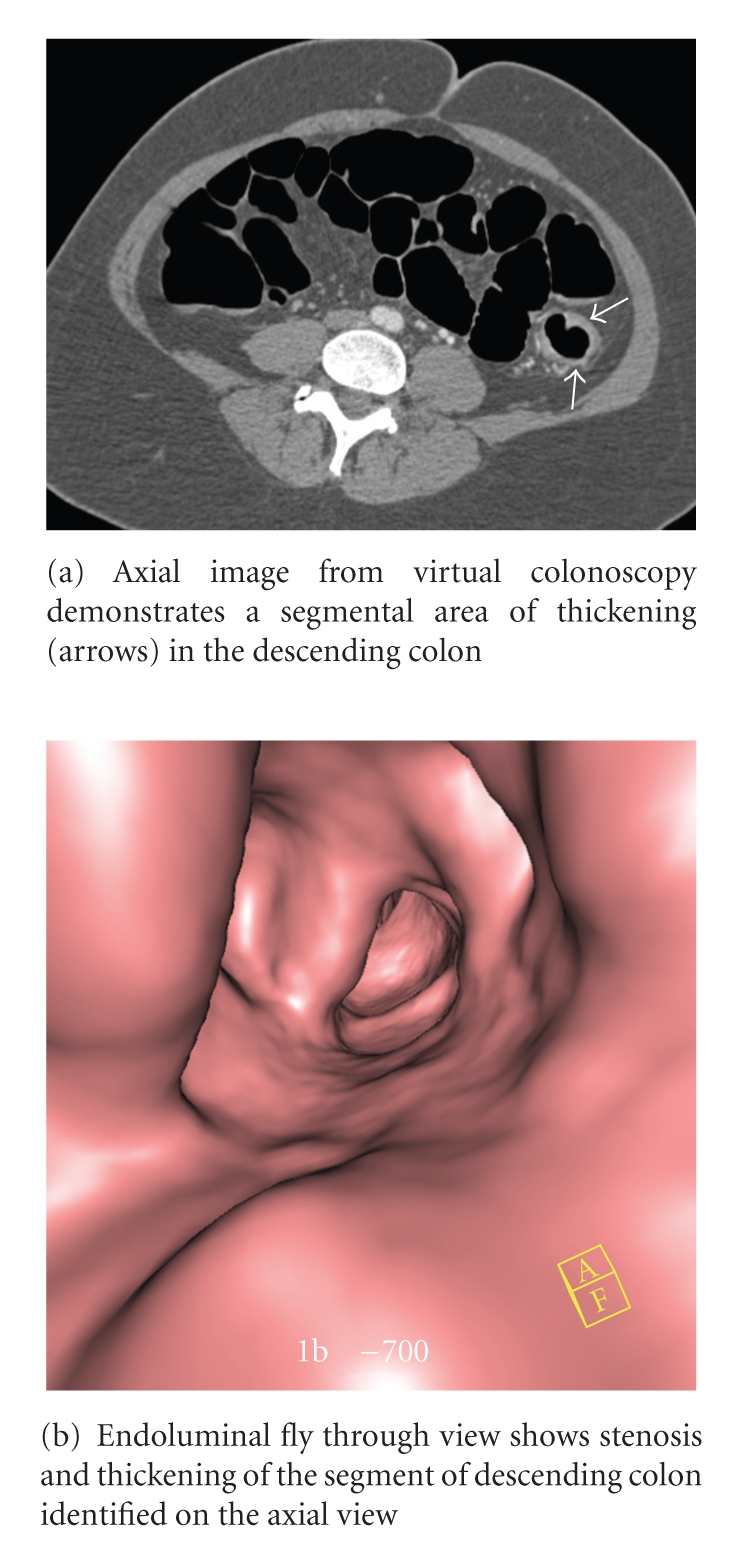


**Figure 3:**
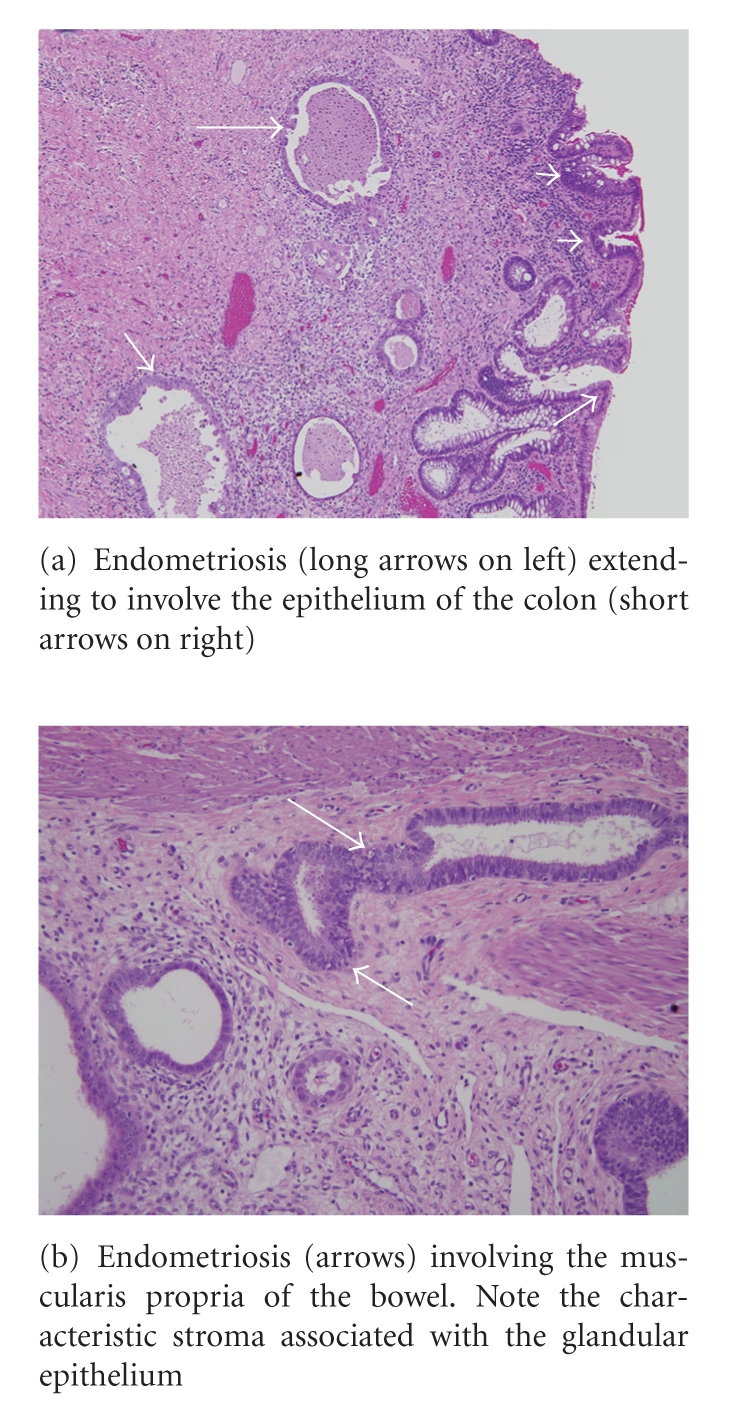


**Figure 4:**
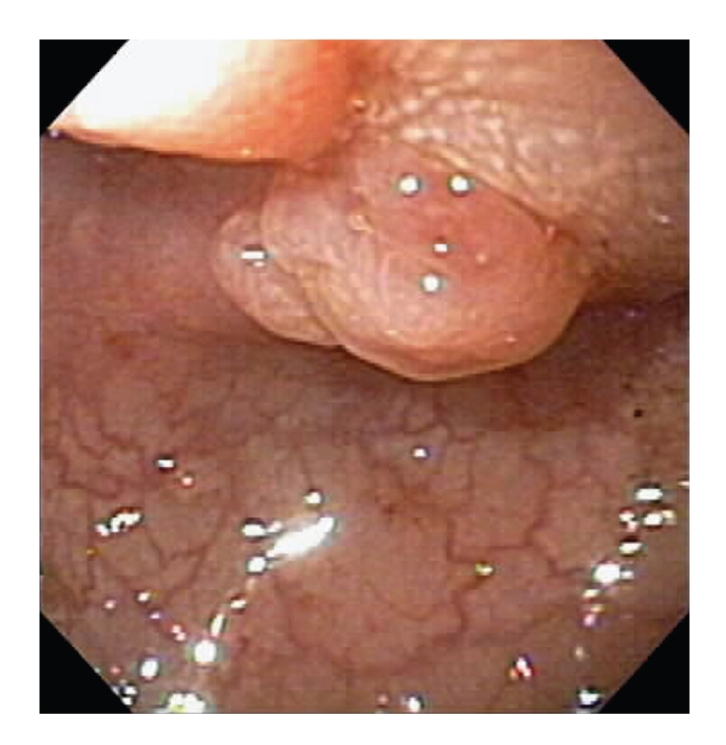
Endoscopy view showing lobular mass in the lumen of the sigmoid colon.
